# Exercise training reduces insulin resistance in postmyocardial infarction rats

**DOI:** 10.14814/phy2.12339

**Published:** 2015-04-23

**Authors:** Youhua Wang, Zhenjun Tian, Weijin Zang, Hongke Jiang, Youyou Li, Shengpeng Wang, Shengfeng Chen

**Affiliations:** 1 Department of Physical Education, Shaanxi Normal UniversityXi'an, Shaanxi, China; 2 Department of Physiology, University of Maryland School of MedicineBaltimore, MD, USA; 3 Department of Pharmacology, Xi'an Jiaotong University, College of Medicine Xi'anShaanxi, China; 4 Department of Physiology and Department of Cardiology, Fourth Military Medical UniversityXi'an, Shaanxi, China

**Keywords:** Aorta, exercise training, insulin resistance, myocardial infarction, signal pathway

## Abstract

Myocardial infarction (MI) induces cardiac dysfunction and insulin resistance (IR). This study examines the effects of MI-related IR on vasorelaxation and its underlying mechanisms, with a specific focus on the role of exercise in reversing the impaired vasorelaxation. Adult male Sprague–Dawley rats were divided into three groups: Sham, MI, and MI+Exercise. MI+Exercise rats were subjected to 8 weeks of treadmill training. Cardiac contraction, myocardial and arterial structure, vasorelaxation, levels of inflammatory cytokines, expression of eNOS and TNF-*α*, and activation of PI3K/Akt/eNOS and p38 mitogen-activated protein kinase (p38 MAPK) were determined in aortas. MI significantly impaired endothelial structure and vasodilation (*P *< 0.05–0.01), as indicated by decreased arterial vasorelaxation to ACh and insulin. MI also attenuated the myocardial contractile response, decreased aortic PI3K/Akt/eNOS expression and phosphorylation by insulin, and increased IL-1*β*, IL-6, and TNF-*α* expression and p38 MAPK activity (*P *< 0.05–0.01). Exercise improved insulin sensitivity in aortas, facilitated myocardial contractile response and arterial vasorelaxation to ACh and insulin, and increased arterial PI3K/Akt/eNOS activity. Moreover, exercise markedly reversed increased p38 MAPK activity and normalized inflammatory cytokines in post-MI arteries. Inhibition of PI3K with LY-294002, and eNOS with L-NAME significantly blocked arterial vasorelaxation and PI3K/Akt/eNOS phosphorylation in response to insulin. In conclusion, these results demonstrate that endothelial dysfunction in response to insulin plays an important role in MI-related IR. The reversal of IR by exercise is most likely associated with normalizing inflammatory cytokines, increasing the activation of PI3K/Akt/eNOS, and reducing the activation of p38 MAPK.

## Introduction

Myocardial infarction leads to cardiac dysfunction, vascular endothelial dysfunction, and other complications such as heart failure and insulin resistance (IR) (Bonora et al. 2007; Robins et al. 2011; Banerjee et al. 2013; McGuire and Gore 2013). Accumulating data indicate that myocardial infarction and IR often coexist, both in humans and in experimental animal models (Gruzdeva et al. 2013a; McGuire and Gore 2013; Vardeny et al. 2013; Trifunovic et al. 2014). Previous studies also demonstrate an independent association between IR and multivessel coronary artery disease in nondiabetic post-MI patients; this finding strengthens the experimental evidence for IR independent of glucose control and other components of the metabolic syndrome (Karrowni et al. 2013). It has been shown that IR in nondiabetic post-MI patients is associated with an increased risk for heart failure (Banerjee et al. 2013; McGuire and Gore 2013; Vardeny et al. 2013). Considerable evidence demonstrates that myocardial infarction induces the release of proinflammatory cytokines and increases p38 mitogen-activated protein kinase (p38 MAPK) activity (Maier et al. 2005; Kompa et al. 2008; Nunes et al. 2008; Krishnamurthy et al. 2009). Evidence also shows that inflammatory cytokines injure endothelial tissue and produce IR (Samaan 2011; Sturek 2011). Furthermore, inflammatory cytokines cross talk with eNOS signaling, which can inhibit endothelial nitric oxide synthase (eNOS) expression, leading to dysfunction in vasorelaxation, whereas increasing the activation of eNOS-mediated NO produces an anti-inflammatory effect (Qiu et al. 2012).

Growing literature implicates IR as a risk factor for the development of heart failure and suggests that measures beyond targeting coronary artery disease are necessary to mitigate this risk (Stakos et al. 2013; Vardeny et al. 2013). In recent years, in an attempt to establish the role of exercise training in IR, studies using both human and animal models have been performed. Experiments using diabetic or overweight patients have revealed that exercise could ameliorate IR (Fisher et al. 2012; Hall et al. 2013; Arciero et al. 2014; de Sousa et al. 2014). Similarly, animal experiments verify that exercise training reversal of hypertensive response to insulin is most likely associated with improved insulin sensitivity in an eNOS-dependent manner and reduced oxidative and nitrative stresses in aging rats (Li et al. 2009). Numerous studies demonstrate that eNOS-derived NO exerts a pivotal role in vasorelaxation and glucose uptake in peripheral insulin-targeted organs (Dimmeler et al. 1999; Ma et al. 2006). Our previous study had indicated that exercise training protects MI-induced injury of peripheral mesenteric arteries via a PI3K/Akt-mediated eNOS-dependent pathway (Wang et al. 2010b). It is unknown whether reduction in MI-induced IR due to exercise training is also a result of the eNOS-dependent pathway. Another study showed that IR has an association with endothelial dysfunction in large, major arteries (Li et al. 2009; Xie and Liu 2009). However, the relationship between IR and incident heart failure is not well established. Neither is it clear whether the relationship between IR and heart failure is mediated entirely by coronary artery disease, or whether other pathways could be involved. In this study, we focus on the aorta to try to explain the relationship between aortic functional changes and post-MI IR.

Therefore, in this study, we sought to determine the relationship between myocardial infarction and IR. Secondary questions included determining whether exercise training reduces IR post-MI, as well as studying the specific signaling mechanism by which exercise training reduces post-MI-related IR.

## Materials and methods

### Drugs and chemicals

LY-294002, NG-nitro-L-arginine methylester (L-NAME), acetylcholine (ACh), insulin, sodium nitroprusside (SNP), phenylephrine (PE), tetrazolium chloride (TTC), and dimethyl sulfoxide (DMSO) were purchased from Sigma (St. Louis, MO, USA). Antibodies against PI-3 kinase p85 subunit, phosphorylated PI-3 kinase p85 subunit (p-PI3K), Akt, phosphorylated-Akt (Ser-473) (p-Akt), endothelial nitric oxide Synthase (eNOS), phosphorylated-eNOS (Ser-1177) (p-eNOS), p38 mitogen-activated protein kinases (p38 MAPK), phosphorylated-p38 MAPK (p-p38 MAPK), IL-1*β*, IL-6, IL-10 and TNF-*α* were obtained from Cell Signalling Technology (Beverly, MA, USA). The NO Assay Kit was from Nanjing Jiancheng Bioengineering Institute (Nanjing, Jiangsu, China). IL-1*β*, IL-10, IL-6, TNF-*α,* and insulin radioimmunoassay kit were from Beijing North Institute of Biological Technology (Beijing, China). Bicinchoninic acid (BCA) protein Assay Kit was from Pierce (Rockford, IL, USA).

### Animal myocardial infarction model

Adult male Sprague–Dawley rats (6-week-old) weighing 200 ± 18 g (purchased from the Experimental Animal Center of Xi'an Jiaotong University, Xi'an, China) were used in this study. All animals were housed individually in a temperature-controlled animal room (22–24°C) under a 12-h light (7:30–19:30)/12-h dark (19:30–7:30) circadian cycle with free access to chow and water. The protocol of MI was created by ligation of the left anterior descending coronary artery (LAD) (Wang et al. 2010b). Briefly, rats were anesthetized with 2% isoflurane mixed with oxygen. After left thoracotomy, the heart was exteriorized and the LAD was ligated approximately 3 mm below the left atrium with a 5–0 silk suture. For the sham group, the suture was removed without tying and no infarction was created. The ST segment of electrocardiogram was elevated in myocardial infarction rats. All experimental procedures and protocols conformed to the recommended guidelines on the care and use of laboratory animals issued by the Chinese Council on Animal Research. The study was approved by the ethical committee of Shaanxi Normal University.

### Exercise protocol

Rats were randomly assigned to the following experimental groups: sham-operated control (Sham, *n* = 30), sedentary myocardial infarction (MI, *n* = 30), and myocardial infarction + exercise (MI + Ex, *n* = 30). Sham and MI animals were housed in separate cages with free access to chow and water; MI + Ex animals were trained on a rodent treadmill 1 week after surgery for 5 days per week subsequently for 8 weeks. Animals that were unable to perform exercise were excluded. Treadmill speed and exercise duration were adapted from previous studies (Wang et al. 2010b). Training was started at 10 m/min, 5° gradient for 10 min per session. Speed and duration were gradually increased to 16 m/min and 50 min per session. The exercise protocol was based on the fact that low-intensity exercise training has no harmful effects on MI patients and improves cardiovascular function. This exercise regimen was well tolerated by rats post-MI. Except for excluded rats, saved rats successfully completed the 8 weeks of exercise training.

#### Oral glucose tolerance test (OGTT) and insulin sensitivity test (IST)

OGTT and IST were performed in 6–8 animals each group following an 8-h fasting. For OGTT, rats were given an oral glucose (2 g/kg), then whole blood glucose levels were determined at 0, 30, 60, 90, and 120 min after glucose challenge. For IST, rats were given an i.p. injection of insulin (0.5 U/kg) (Li et al. 2009), and whole blood glucose levels were determined at 0, 30, 60, 90, 120, and 240 min after insulin injection using tail clipping. Values were normalized to the initial glucose levels prior to initiation of the IST test.

### Hemodynamic study, TTC staining, and measurement of insulin and inflammatory cytokines in serum and NO in arteries

At the end of 8 weeks, 6–8 animals in each group were anesthetized with sodium pentobarbitone (30 mg/kg, iv). Body temperature was maintained and all measurements were taken in the anesthetized state under basal conditions. The right femoral artery was cannulated and arterial blood pressure was measured via a polyethylene catheter. Arterial systolic blood pressure (SBP) and diastolic blood pressure (DBP) were continuously monitored via a data acquisition system (Powerlab/4SP; AD Instruments, Bella Vista, NSW, Australia). Another heparin-filled polyethylene catheter (PE-50) was inserted into the left ventricle through the right carotid artery to allow for measurement of heart rate (HR), left ventricular systolic pressure (LVSP), and left ventricular end diastolic pressure (LVEDP). Positive and negative maximal values of the instantaneous first derivative of left ventricular pressure (± d*P*/d*t* max) were calculated. After 15–20 min of stabilization, arterial blood pressure was continuously monitored. After recording hemodynamic measurements, the chest was opened and the heart with the aortic root was carefully removed, washed in PBS, perfused from the aortic root with PBS, and then perfused with 2 mL 5% TTC. The heart was then placed into liquid nitrogen for 1 min and then quickly cut into 3–5 mm sections with a razor blade. After TTC staining, the area of infarction exhibited a white color, whereas noninfarcted areas were red.

Blood samples were taken from the abdominal aorta of rats and centrifuged at 2500 rpm for 20 min at 4°C, and then serum was transferred to a fresh tube. Serum levels of insulin and inflammatory cytokines (IL-1*β*, IL-10, IL-6, and TNF-*α*) were determined using radioimmunoassay kits. The aortas were treated with insulin (1.5 × 10^−6^ mol/L) for 15 min, and in selected experiments, aortas were preincubated with LY-294002 (20 *μ*mol/L) or L-NAME (0.5 mmol/L) for 15 min; thereafter, aortas were homogenized in 0.9% NaCl solution (1:10, wt/vol), and centrifuged at 3000 rpm for 15 min. The pellet was discarded. NO concentrations in the supernatant were quantified by a NO detection kit (Nitrate Reductase). All experiments were performed in accordance with the manufacturer's instructions.

### Preparation of artery rings and isometric tension measurement

Aortic rings were prepared after hemodynamic measurements. The abdominal aortas of 6–8 rats in each group were gently isolated and immersed immediately in cold oxygenated Krebs solution (119 mmol/L NaCl, 4.7 mmol/L KCl, 2.5 mmol/L CaCl_2_, 1 mmol/L MgCl_2_, 25 mmol/L NaHCO_3_, 1.2 mmol/L KH_2_PO_4_, and 11 mmol/L D-glucose). Arteries were carefully cleaned of fat and connective tissue and cut into 3-mm-length rings under stereo microscope. A part of the arteries were removed and saved to assess NO levels and perform western blot experiments. Endothelium was removed by gentle scraping with a stainless steel wire inserted into the artery. Removal of the endothelium was confirmed by a lack of relaxation in response to 1 *μ*mol/L ACh. Isometric tension was measured as previously described (Zhao et al. 2013). The artery rings were mounted on two L-shaped stainless steel holders, one of which was fixed to the organ bath and the other was connected to a force displacement transducer (Beijing Aeromedicine Engineering Research Institute, Beijing, China) attached to a Taimeng BL-420F biotic signal collection and analysis system (Taimeng Instruments Co., Chengdu, China) for continuous recording of isometric tension. The mounted artery rings were immersed in organ chambers containing Krebs solution of 1 mL, which was continuously gassed with carbogen (95% air and 5% CO2) at pH 7.4 and maintained at 37°C. The resting tension was adjusted to 2.5 g, an optimal tension that was determined previously in length–active tension relationship experiments. After mounting with a previously determined optimal resting tension for 60 min, each ring was first contracted by 10 *μ*mol/L PE and then challenged with 1 *μ*mol/L ACh to confirm the vessel's contractility and the integrity of its endothelium. The rings were then washed to restore tension to baseline and allowed to stabilize for 60 min. Thereafter, the rings were preconstricted with PE (0.1–2 *μ*mol/L) to comparable constriction levels in each group, and relaxant responses to cumulative doses of ACh, insulin, and sodium nitroprusside (SNP) were assessed. Cumulative concentration–response curves to ACh, SNP, PE or insulin were obtained in aorta rings. The PI3K-specific inhibitor, LY-294002, was dissolved in DMSO and diluted in saline containing 0.1% DMSO. In another experiment, aorta rings were incubated for 15 min with inhibitors LY-294002 (20 *μ*mol/L) or L-NAME (0.5 mmol/L) before being stimulated with ACh and insulin in order to study the effects of the blockade of PI3K or eNOS, respectively, on ACh and insulin-induced vascular relaxation, using insulin dosage based on a previous study (Li et al. 2009). During the resting period, the bath solution was replaced every 15 min.

### Masson's trichrome staining, hematoxylin and eosin staining, immunohistochemistry, and transmission electron microscopy

Six rats from each group were anesthetized with sodium pentobarbital (30 mg/kg, i.v.). Rat hearts and abdominal aortas were carefully isolated and fixed with 4% paraformaldehyde for <48 h. Fixed heart and aorta segments were dehydrated and embedded in paraffin, and 5 *μ*m sections were cut with a Leica RM-2162 (Leica, Bensheim, Germany) for Masson's trichrome staining, hematoxylin and eosin staining and immunohistochemistry for eNOS and TNF-*α*. Immunohistochemical detection of eNOS and TNF-*α* was performed as previously described (Wang et al. 2010a,b). For electron microscopic examination, the sections of aortas were cut and fixed by immersion in 2.5% glutaraldehyde in 0.1 mol/L cacodylate buffer (pH 7.4) for 3 h. After washing in cacodylate buffer, tissue was postfixed in 1% OsO4 buffered with 0.1 mol/L sodium cacodylate. The tissue was dehydrated in an ethanol series, infiltrated with propylene oxide and embedded in Epon 812. Toluidine blue-stained sections (1 *μ*m) were examined by light microscopy, and appropriate areas of tissue were selected for cutting thin sections (LKB-NOVA, Sweden). Thin sections were stained with uranyl acetate and lead citrate and examined using an electron microscope (JEM-2000EX, Filgen, Nagoya, Japan).

### Western blotting

Protein expression and phosphorylation were measured using western blot as previously described (Wang et al. 2010b). Total proteins from aortic tissue homogenates (4–6 rats each treatment) were quantified with the BCA protein assay and separated in SDS-PAGE gels, transferred to a polyvinylidene difluoride (PVDF) membrane, and blocked with 5% nonfat dry milk. Immunoblots were incubated with primary antibodies overnight at 4°C followed by incubation with the corresponding secondary antibodies at room temperature for 1 h. After immunoblots of phospho-PI3K, phospho-Akt, phospho-eNOS, and phospho-p38 MAPK, PVDF membranes were stripped with stripping buffer at 50°C for 30 min and reprobed for total PI3K, Akt, eNOS, p38 MAPK, IL-1*β*, IL-10, and TNF-*α*. Determination of PI3K, Akt, eNOS, and p38 catalytic activities in intact aorta was performed as previously described (Wang et al. 2010b). Aortas were treated with insulin (1.5 × 10^−6^ mol/L) (Li et al. 2009) for 15 min in the presence or absence of either LY-294002 (20 *μ*mol/L) or L-NAME (0.5 mmol/L) for 15 min. *β*-actin was used as the internal loading control. The blots were visualized with ECL-plus reagent. The immunoblot bands were quantified by LabImage version 2.7.1 (Kapelan GmbH, Halle, Germany).

### Statistical analysis

For semiquantification of the average optical density (OD) of eNOS and TNF-*α* in left ventricular myocardium, the method was performed as in our previous study (Wang et al. 2010a,b). Images of the stained sections were acquired and analyzed with Image Pro Plus 5.1, a computer-assisted image analysis system. The activation of PI3K, Akt, eNOS, and p38 MAPK was calculated by comparing the ratios of phosphorylated protein intensity to total protein intensity in each of the groups. Data are presented as the mean ± S.E.M. Student's *t*-test or analysis of variance (ANOVA) was used to assess significance of differences in all experimental data. When ANOVA revealed significant differences, Tukey's post hoc test was used for multiple comparisons. *P *<* *0.05 was considered significant. All statistical calculations were performed using a commercially available software package (SPSS, Version 15.0, Inc., Chicago, IL).

## Results

### Myocardial infarction induces significant systemic IR

**Figure 1:**
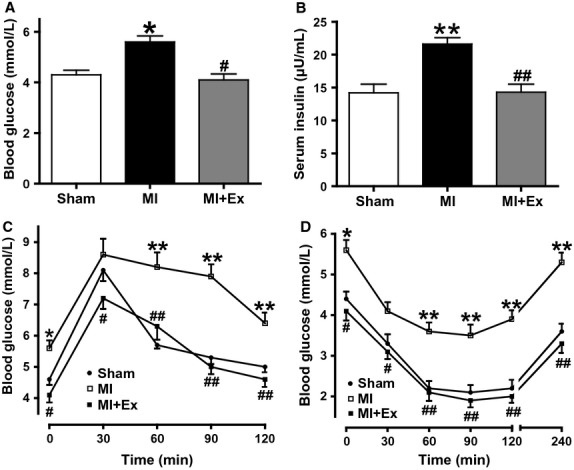
The levels of fasting blood glucose and insulin, oral glucose tolerance test (OGTT), and insulin sensitivity test (IST) in rats. (A) Blood glucose levels after 8 h fasting; (B) Serum insulin levels after 8 h fasting. After an 8 h fasting, rats were given oral glucose (2 g/kg) for OGTT (C) or an injection (i.p.) of insulin (0.5 U/kg) for IST (D). Whole-blood glucose levels were measured at the indicated time points. The levels of blood glucose and insulin were markedly elevated in MI rats (*P *<* *0.05–0.01). Exercise training reduces the levels of the blood glucose and insulin (*P *<* *0.05–0.01). Data are expressed as mean ± SEM, *n *=* *6–8 per group. *P <* *0.05, **P <* *0.01, vs. Sham; ^#^*P *<* *0.05, ^##^*P *<* *0.01 vs. MI.

ANOVA analysis results showed a significant difference in blood glucose and serum insulin exist among three groups. Then pairwise comparisons were performed between MI and Sham, MI + Ex and MI, Sham and MI + Ex (*P *<* *0.05–0.01). In myocardial infarction rats, levels of fasting blood glucose and serum insulin were significantly higher compared with those in Sham rats (*P *<* *0.05, *P *<* *0.01, respectively) (Fig.1A and B). Moreover, blood glucose levels in post-MI rats were significantly higher than those in Sham rats within the entire 120 min observation period after OGTT. (*P *<* *0.05–0.01) (Fig.1C). Whole-body insulin sensitivity assessed by blood glucose reductions in response to insulin was markedly attenuated in post-MI rats compared with those in Sham ones (Fig.1D). Exercise training decreased blood glucose and serum insulin almost to the levels of Sham rats. These results indicate that systemic IR is present in MI rats used in this study.

### Effects of myocardial infarction and exercise training on infarction size, animal characteristics, hemodynamic parameters, and inflammatory cytokine levels

**Figure 2:**
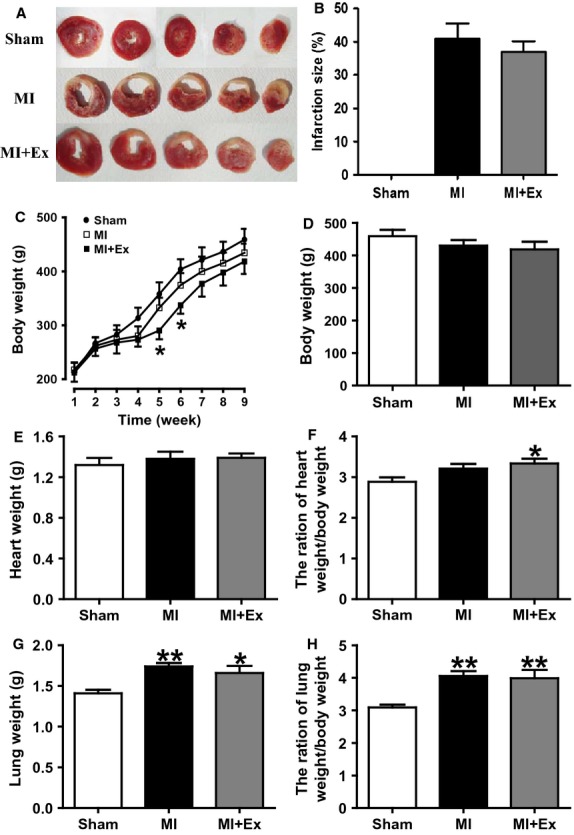
Effects of myocardial infarction and exercise training on animal characteristics. MI + Ex have a trend reduced infarction size and body weight, and elevated the ratio of heart to body weight. MI and MI + Ex rats both had significantly elevated lung weight and the ratio of lung weight to body weight (*P *<* *0.05–0.01). Data are expressed as mean ± SEM, *n* = 6–8 per group. *P <* *0.05, **P <* *0.01, vs. Sham; ^#^*P *<* *0.05, ^##^*P *<* *0.01 vs. MI.

**Figure 3:**
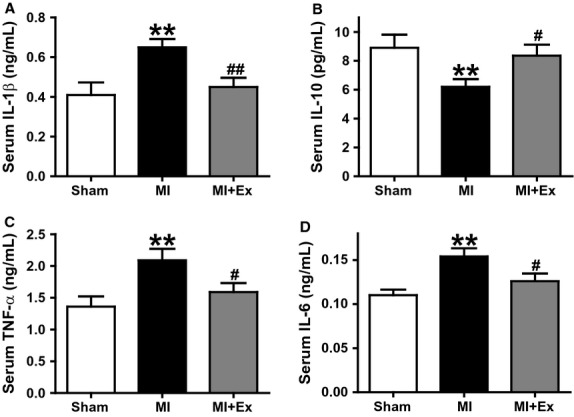
Effects of myocardial infarction and exercise training for the concentration in serum of IL-1*β*, IL-10, IL-6, and TNF-*α*. The levels of IL-1*β* and TNF-*α* were markedly elevated in MI rats, and the levels of IL-10 and IL-6 were markedly decreased (*P *<* *0.05–0.01), whereas exercise training restored the expression of IL-1*β*, IL-10, IL-6, and TNF-*α* in the aorta. Data are expressed as mean ± SEM, *n* = 6–8 per group. *P <* *0.05, **P <* *0.01 vs. Sham; ^#^*P *<* *0.05, ^##^*P *<* *0.01 vs MI.

The results of ANOVA analysis showed significant difference among three groups with regard to animal characteristics, hemodynamic parameters, and inflammatory cytokines (*P *<* *0.05–0.01). Then pairwise comparisons were performed between MI and Sham, MI + Ex and MI, Sham and MI + Ex. Results showed that ligation for 8 weeks induced myocardial infarction that occupied an average of 41% of cardiac tissue, and exercise partly but not significantly reduces myocardial infarction (Fig.2A and B). The general conditions of all animals were observed and recorded throughout the study. From the second week, rats in the MI group and MI + Ex group had a significantly lower increase in body weight than that in the Sham groups. This difference was especially significant at weeks 5 and 6 (*P *<* *0.05) (Fig.2C). However, at the end of 9 weeks, there was no significant difference in body weight within the three groups (Fig.2C and D). As shown in Figure2E–H, there is also no difference in heart weight, but MI + Ex elevated the ratio of heart to body weight. Moreover, MI and MI + Ex rats both had significantly elevated lung weight and elevated ratio of lung weight to body weight (*P *<* *0.05–0.01). These results demonstrated that although MI did not induce cardiac hypertrophy, it induced pulmonary compensatory increases in weight. Hemodynamic parameters were the same as shown previously (Wang et al. 2010b). MI elevated heart rate, whereas SBP, DBP, LVSP, and ±d*P*/d*t* max were markedly attenuated. LVEDP was increased compared with sham (*P *<* *0.05–0.01), indicating that 8 week ligation caused evident cardiac dysfunction. Exercise for 8 weeks significantly ameliorated cardiac dysfunction induced by MI, as evidenced by increased SBP, LVSP and ±d*P*/d*t*, and decreased LVEDP (*P *<* *0.05–0.01 vs. MI) (data not shown). In addition, we measured the content of inflammatory cytokines in serum. As shown in Figure3A–D, MI elevated the contents of Serum IL-1*β* and TNF-*α*, and decreased IL-10 levels significantly compared with Sham ones (*P *<* *0.05–0.01). The concentrations of IL-1*β* and TNF-*α* were markedly lower, whereas IL-10 level was significantly higher in the MI + Ex group than in the MI group (*P *<* *0.05–0.01) (Fig.3A–D).

### Effects of myocardial infarction and exercise training on structural changes of left ventricular myocardium and aortic endothelial cells, and expression and distribution of eNOS and TNF-*α*

**Figure 4:**
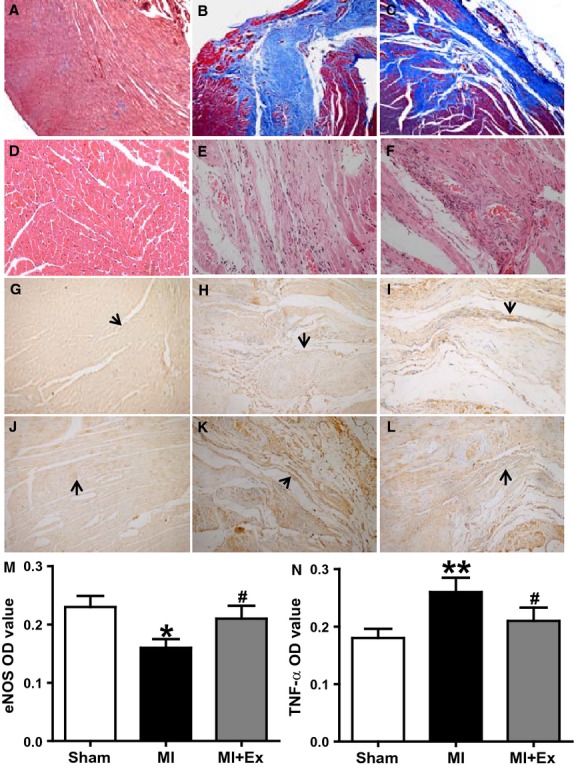
Effects of myocardial infarction and exercise training on myocardial fibrosis and the expression of eNOS and TNF-*α* in the infarct border zone of left ventricular myocardium. Masson's trichrome staining (myocardial tissue: red color, fibrosis: blue color) and hematoxylin and eosin staining (myocardial tissue: red color, fibrosis: pink color) showed that MI-induced fibrotic changes in the infarct border zone within left ventricular myocardium; this increase in fibrosis was attenuated in the MI + Ex groups. The expression of TNF-*α* was markedly elevated and the expression of eNOS was markedly decreased in MI rats (*P *<* *0.05–0.01), whereas exercise training increased the expression of eNOS and decreased the expression of TNF-*α* in the infarct border zone of left ventricular myocardium (*P *<* *0.05–0.01). Sham: A ×10, D, G, and J ×40; MI: B ×10, E, H and K ×40; MI + Ex: C ×10, F, I and L ×40, all cross section. (A, B, C) Masson's trichrome staining; (D, E, F) Hematoxylin and eosin staining. (M) Average optical density value for eNOS; (N) Average optical density value for TNF-*α*. Data are expressed as mean ± SEM, *n* = 6 per group. *P <* *0.05, **P <* *0.01, vs. Sham; ^#^*P *<* *0.05, ^##^*P *<* *0.01 vs. MI.

**Figure 5:**
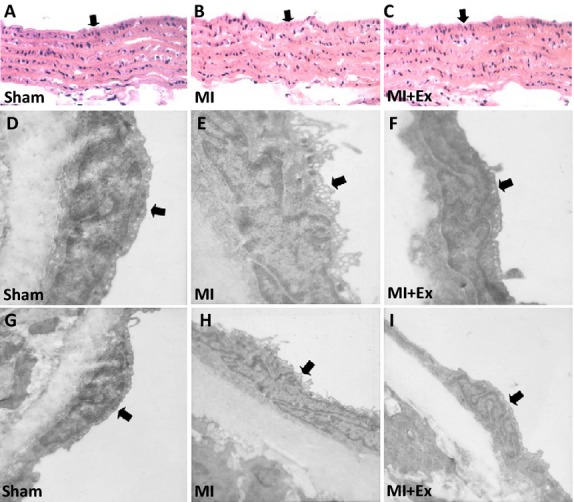
Effects of myocardial infarction and exercise training on microstructure and ultrastructure of aortic endothelial cells. Microscopy and transmission electron microscopy showed that endothelial cells in the Sham group exhibited a normal structure. However, MI-induced injury in endothelial structure, and exercise training restored the endothelial structure integrity. (A, B and C) Hematoxylin and eosin staining for aortas (longitudinal section); (D, E, F, G, H and I) Transmission electron microscopy for aortas (cross section). Sham: A ×200, D ×10,000, G ×5000, MI: B ×200, E ×10,000, F ×5000; MI + Ex: C ×200, F ×10,000, I ×5000.

As shown in Figure4A–F, Masson's trichrome staining (myocardial tissue: red color, fibrosis: blue color) and hematoxylin and eosin staining (myocardial tissue: red color, fibrosis: pink color) showed that MI-induced fibrosis changes in the infarct border zone within left ventricular myocardium (Fig.4B and E); this increase in fibrosis was attenuated in the MI + Ex groups (Fig.4C and F). The structural integrity of the vascular endothelium plays a critical role in vascular homeostasis. Therefore, we assessed the structure of aortic endothelial cells with normal microscopy and transmission electron microscopy. As shown in Figure5A, D, and G, endothelial cells in the Sham group exhibited a normal structure. However, an abnormal endothelial structure was observed in the MI group (Fig.5B, E, and F); some endothelial cells were edematous, resulting in a rough cell surface, and some endothelial cells had cytoplasm containing clear vacuoles, and the membrane of the endothelial cells was not intact. The internal elastic membrane of the aorta was ruptured, and in some areas smooth muscle layers were directly exposed to the vessel lumen due to endothelial cell desquamation. The endothelial injury was reduced after treatment with exercise training (Fig.5C, F and I). These results indicate structural degradation of endothelium, which may be a contributor to dysfunctional vasorelaxation observed in aortas from post-MI rats. Exercise training can protect structure and restore integrity of aortic endothelium.

**Figure 6:**
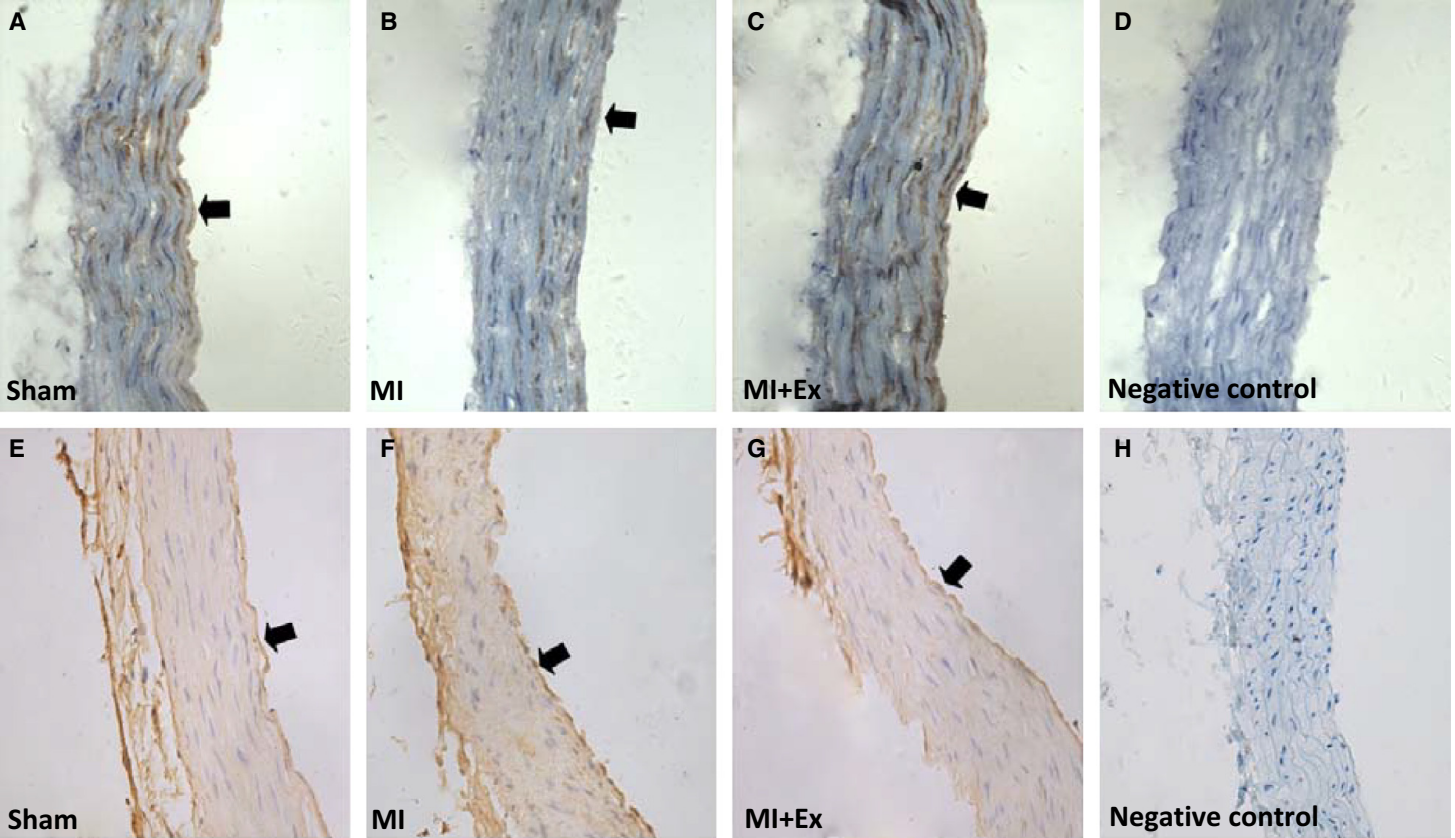
Effects of myocardial infarction and exercise training on the expression and distribution of eNOS and TNF-*α* in the aorta. The eNOS and TNF-*α* were mainly expressed within endothelial cells of the aorta. Sham: A, E ×200; MI: B, F ×200; MI + Ex: C, G×200; Negative control: D, H ×200, all cross section.

In addition, we assessed the expression and distribution of eNOS and TNF-*α* in left ventricular myocardial tissue. Immunohistochemistry showed that eNOS and TNF-*α* are expressed in three groups (Fig4G–L). ANOVA analysis showed a significant difference in the OD of eNOS and TNF-*α* among three groups (*P *<* *0.05–0.01). Then pairwise comparisons were performed between MI and Sham, MI + Ex and MI, Sham and MI + Ex. MI increased the expression of TNF-*α* and decreased the expression of eNOS in the left ventricular myocardial tissue (Fig.4H, K, M and N). Eight weeks of exercise training increased the expression of eNOS and decreased the expression of TNF-*α* compared with MI (Fig.4I, L, M and N). At the same time, we also assessed the expression and distribution of eNOS and TNF-*α* in aortas. We observed that eNOS and TNF-*α* was expressed in all three groups, and mainly within endothelial cells (Fig.6).

### Effects of myocardial infarction and exercise training on aortic vasodilatations

**Figure 7:**
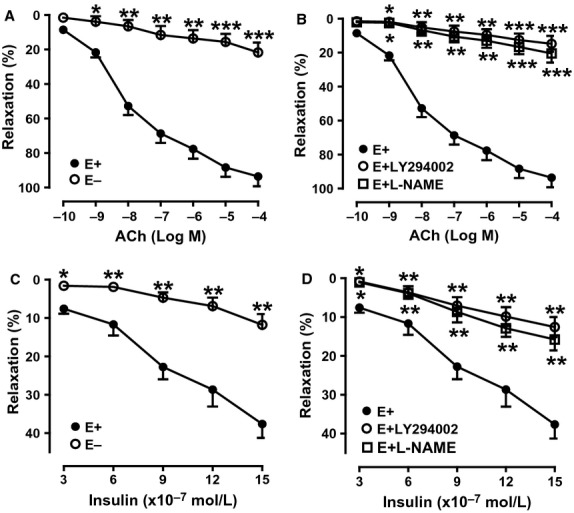
Dose-dependent vasorelaxation of PE preconstricted artery rings in response to ACh and insulin. (A) Dose-dependent vasorelaxation of PE preconstricted artery rings in response to ACh ranging from 10^−10^ to 10^−4^ mol/L in endothelium-intact and endothelium-denuded arteries in Sham rats; (B) Effects of LY29400 and L-NAME on dose-dependent vasorelaxation of PE preconstricted artery rings in response to ACh ranging from 10^−10^ to 10^−4^ mol/L in endothelium-intact of Sham rat arteries; (C) Dose-dependent vasorelaxation of PE preconstricted artery rings in response to insulin ranging from 3 to 15 × 10^−7^ mol/L in endothelium-intact and endothelium-denuded Sham arteries; (D) Effects of LY29400 and L-NAME on dose-dependent vasorelaxation of PE preconstricted artery rings in response to insulin ranging from 3 to 15 × 10^−7^ mol/L in endothelium-intact of Sham arteries. Either LY29400 (20 *μ*mol/L) was or L-NAME (0.5 mmol/L) was added to perfusate 15 min before the experiments. E+ endothelium-intact; E− endothelium-denuded. Data are expressed as mean ± SEM, *n *=* *8–10 rings/group from 6–8 rats. *P <* *0.05, **P <* *0.01, ***P <* *0.001 versus endothelium-intact artery rings.

**Figure 8:**
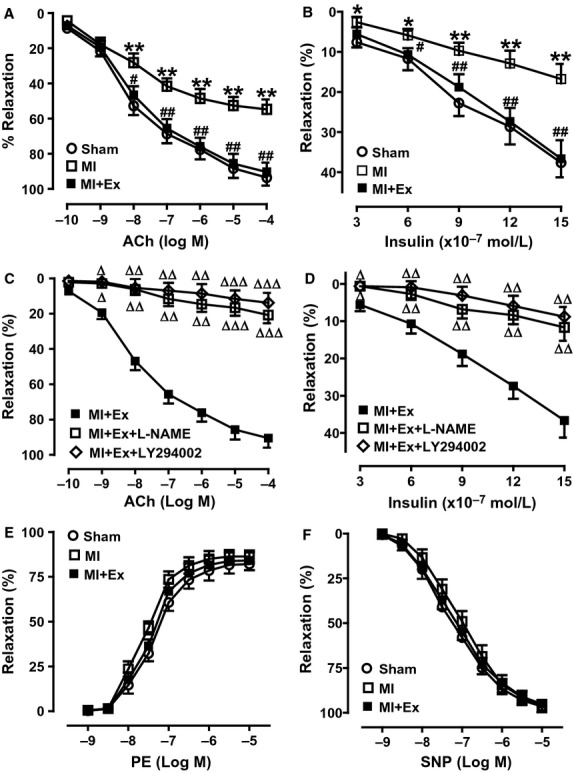
Effects of myocardial infarction and exercise training on dose-dependent vasorelaxation of PE preconstricted artery rings in response to ACh, SNP, and insulin; constriction in response to phenylephrine (PE). (A) Effects of myocardial infarction and exercise training on dose-dependent vasorelaxation of PE preconstricted artery rings from three groups of rats in response to ACh ranging from 10^−10^ to 10^−4^ mol/L; (B) Effects of myocardial infarction and exercise training on vasorelaxation to insulin ranging from 3 × 10^−7^ to 15 × 10^−7^ mol/L; (C) Inhibition of PI3K and eNOS attenuated the effects of exercise on dose-dependent vasorelaxation of PE preconstricted artery rings from MI + Ex rats to ACh ranging from 10^−10^ to 10^−4^ mol/L; (D) Inhibition of PI3K and eNOS attenuated the effects of exercise on vasorelaxation to insulin ranging from 3 × 10^−7^ to 15 × 10^−7^ mol/L; (E) Effects of myocardial infarction and exercise training on dose-dependent constriction in response to phenylephrine (PE) ranging from 10^−9^ to 10^−5^ mol/L. (F) Effects of myocardial infarction and exercise training on dose-dependent vasorelaxation in response to SNP ranging from 10^−9^ to 10^−5^ mol/L. Data are expressed as mean ± SEM, *n *=* *8–10 rings/group from 6–8 rats. *P <* *0.05, **P <* *0.01, ***P <* *0.001 versus Sham. ^#^*P *<* *0.05, ^##^*P *<* *0.01 vs. MI. ^Δ^*P *<* *0.05, ^ΔΔ^*P *<* *0.01, ^ΔΔΔ^*P *<* *0.001 vs. MI + Ex.

Previous evidence showed that IR has a close relationship with endothelial dysfunction. In the present study, we isolated aortas from the three group's animals and measured ACh and insulin-induced vasorelaxation. ANOVA analysis results showed a significant difference in ACh and insulin-mediated vasorelaxation between E+ and E− in Sham aortas (*P *<* *0.05–0.001), and among three groups (*P *<* *0.05–0.001), whereas, PE-mediated vasoconstriction and SNP-mediated vasorelaxation had no significant difference among the three groups. The arteries of endothelium-intact in Sham rats were treated with ACh and insulin respectively, resulting in maximal vasorelaxation of 93.6 ± 4.8% and 37.67 ± 3.6%. The mechanical denudation of endothelium almost abolished ACh and insulin-induced vasorelaxation, resulting in maximal vasorelaxation of 16.8 ± 4.6% and 11.8 ± 2.8% (Fig.7A and C). Pretreatment with either LY-294002 or L-NAME both markedly attenuated ACh (the maximal vasorelaxation: 14.8 ± 4.6% and 20.5 ± 5.4%) and insulin-(the maximal vasorelaxation: 12.6 ± 2.6% and 15.8 ± 2.8%) induced vasorelaxation (Fig.7B and D). This indicates that relaxation is an endothelium-dependent process. The endothelium-dependent vasodilator acetylcholine (ACh) and the endothelium-independent vasodilator sodium nitroprusside (SNP) were used to induce vasodilation. The maximum relaxation in response to cumulative additions of ACh was significantly impaired in aortas from the MI group compared with the Sham group (Fig.8A). Exercise training treatment corrected endothelial dysfunction as evidenced by enhanced maximum relaxation in response to ACh almost to the levels of Sham rats in the MI + Ex group (Fig.8A). However, vessels displayed similar vasodilation response to SNP among each group (Fig.8F). Removal of the endothelium did not significantly modify SNP-induced vasorelaxation (data not shown).

To further determine whether MI-induced endothelial dysfunction may affect the endothelial response to insulin, we further measured the vasorelaxation of arterial rings to insulin. As expected, in PE precontracted arterial rings from rats, insulin produced a dose-dependent vasorelaxation with the maximal response occurring at 1.5 × 10^−6^ mol/L insulin (37.7 ± 3.6%) (Fig.8B and D). We also assessed the vasorelaxation of mesenteric arteries in response to insulin, which was similar to the aortic response (data not shown). The dose-dependent vasoconstrictor response of arterial rings to PE was not significantly different among three groups (Fig.8E).

### Effects of myocardial infarction and exercise training on the expression of IL-1*β*, IL-10, IL-6, and TNF-*α*

**Figure 9:**
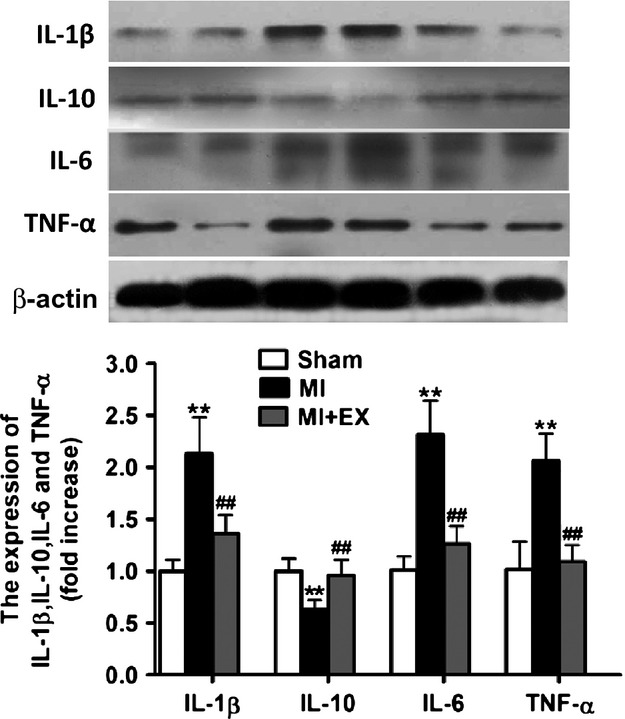
Effects of myocardial infarction and exercise training on the expression of IL-1*β*, IL-10, IL-6, and TNF-*α* in the aorta. IL-1*β* and TNF-*α* expression were markedly upregulated in MI rats, and IL-10 and IL-6 expression were markedly downregulated (*P *<* *0.05–0.01), whereas exercise training restored the expression of IL-1*β*, IL-10, IL-6, and TNF-*α* in aortic arteries. Data are expressed as mean ± SEM, *n *=* *4–6 for each. *P <* *0.05, **P <* *0.01 versus Sham; ^#^*P *<* *0.05, ^##^*P *<* *0.01 versus MI.

Previous studies showed that inflammatory cytokines play a crucial role in the pathogenesis of IR (Sturek 2011). Our present studies measured the expression of inflammatory cytokines on aortas. The results of ANOVA analysis showed a significant difference in IL-1*β*, IL-6, IL-10 and TNF-*α* among three groups (*P *<* *0.05–0.01). Then pairwise comparisons were performed between MI and Sham, MI + Ex and MI, Sham and MI + Ex. As shown in Figure9, there was elevated expression of IL-1*β*, IL-6, and TNF-*α*, and decreased expression of IL-10 in the aortas of MI rats compared with Sham (*P *<* *0.05–0.01). Eight-week exercise training markedly decreased the expression of IL-1*β*, IL-6, and TNF-*α* and increased the expression of IL-10 significantly in Sham rats compared to that in the MI group (*P *<* *0.05–0.01) (Fig.9). There is no significant difference between the Sham and MI + Ex groups. These results show that exercise training normalizes levels of MI-induced inflammatory cytokines, which may be a reason why exercise reduces IR.

### Effects of myocardial infarction and exercise training on the activation of PI3K, Akt, eNOS-NO, and p38 MAPK signaling

**Figure 10:**
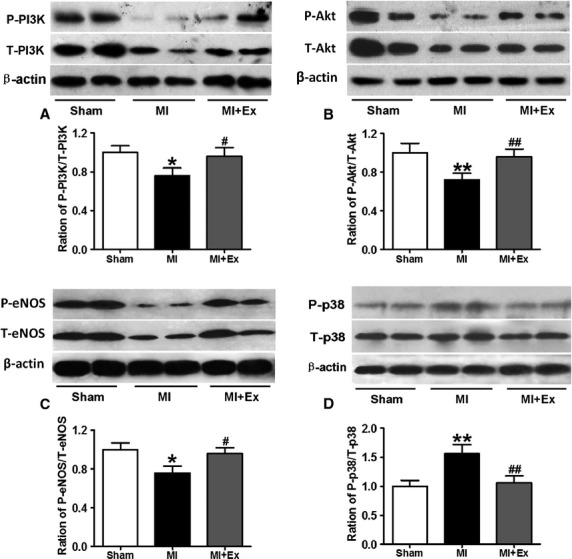
Effects of MI and exercise training on the expression and activation of PI3K (A), Akt (B), eNOS (C), and p38 MAPK (D) in the aorta. MI decreased the expression and the activity of PI3K/Akt/eNOS and increased the activation of p38 MAPK in aortas (*P *<* *0.05–0.01); whereas exercise increased arterial PI3K/Akt/eNOS activity and reduced p38 MAPK activity (*P *<* *0.05–0.01). Data are expressed as mean ± SEM, *n *=* *4–6 for each. *P <* *0.05, **P <* *0.01 versus Sham, ^#^*P *<* *0.05, ^##^*P *<* *0.01 versus MI.

**Figure 11:**
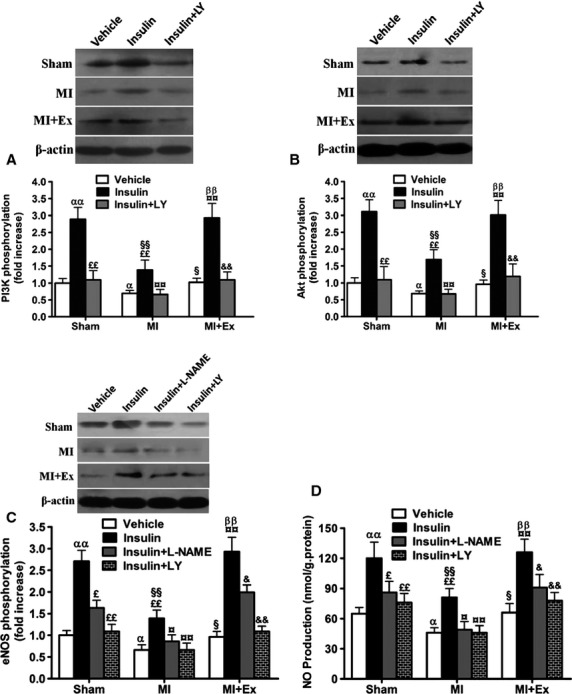
Effects of myocardial infarction and exercise training on the activation of PI3K/Akt/eNOS signaling by insulin treatment of aortas. MI attenuated PI3K/Akt/eNOS phosphorylation and NO production by insulin (*P *<* *0.05–0.01). Exercise training restored PI3K/Akt/eNOS phosphorylation and NO production by insulin (*P *<* *0.05–0.01). Either LY-294002 or L-NAME significantly blocked arterial PI3K/Akt/eNOS phosphorylation and NO production in response to insulin (*P *<* *0.05–0.01). The concentration of insulin was 1.5 × 10^−6^ mol/L. LY-294002 (LY; 20 *μ*mol/L) and L-NAME (0.5 mmol/L) were added to perfusate 15 min before the experiments. Data are expressed as mean ± SEM, *n *=* *4–6 for each. ^α^*P *<* *0.05, ^αα^*P *<* *0.01 versus Sham aortas receiving vehicle. ^££^*P *<* *0.01 versus Sham aortas receiving insulin. ^§^*P *<* *0.05, ^§§^*P *<* *0.01 versus MI aortas receiving vehicle. ^¤¤^*P *<* *0.01 versus MI aortas receiving insulin. ^ββ^*P *<* *0.01 versus MI + Ex receiving vehicle. ^&^*P *<* *0.05, ^&&^*P *<* *0.01 versus MI + Ex receiving insulin.

Considerable evidence has demonstrated that eNOS-derived NO exerts a pivotal role in vasorelaxation, and PI3K and Akt are known to be important upstream regulators of eNOS activation (Fulton et al. 1999; Morello et al. 2009). ANOVA analysis results showed a significant difference in that the activation of PI3K, Akt, and eNOS between each group and each treatment (*P *<* *0.05–0.01). Then pairwise comparisons were performed between MI and Sham, MI + Ex and MI, Sham and MI + Ex. In addition, the pairwise comparisons were done for among each treatment. As shown in Figure10A, B, and C, MI significantly decreased the activation of PI3K and Akt as shown by the p-PI3K to total-PI3K ratio, and p-Akt to total Akt ratio compared with Sham (*P *<* *0.05–0.01), suggesting that MI caused a significant impairment in PI3K and Akt signaling in aortas, which possibly contributes to decreased activation of eNOS-NO signaling. Following 8 weeks of exercise training, phosphorylation of PI3K, Akt, and eNOS, and production of NO were significantly increased in MI + Ex aortas almost to the levels of Sham ones (*P *<* *0.05–0.01) (Fig.10A–C). These results suggest that MI causes a significant impairment of PI3K/Akt/eNOS signaling, and that exercise markedly reverses MI-associated aortic PI3K/Akt/eNOS signaling dysfunction. Previous study showed that p38 MAPK activation upregulates proinflammatory pathways and plays a role in IR (Brown et al. 2015). Therefore, we measured the activation of p38 MAPK; the results show that MI significantly increases p38 MAPK activation, whereas exercise training decreases p38 MAPK activation (Fig.10D). PI3K/Akt pathway has been clearly reported to play a critical role in insulin's metabolic modulation (Zhang et al. 2009). However, the mechanisms involved in the role of insulin in PI3K/Akt signaling pathway post-MI remain largely unclear. The evaluation of vessels stimulated with insulin from each group could provide more convincing evidence that alteration of PI3K/Akt-mediated eNOS signaling pathways contributes to insulin response. Therefore, we further studied protein phosphorylation in the presence of insulin in aortas. Our results showed that MI attenuates PI3K, Akt, eNOS phosphorylation, and NO production induced by insulin compared with Sham (Fig.11A–D). Eight-week exercise restores post-MI-associated loss of PI3K, Akt, eNOS phosphorylation and NO production almost to the levels of Sham rats in response to insulin stimulation (Fig.11A–D). There is no significant difference between Sham and MI + Ex groups. The inhibition of either PI3K by LY-294002 (20 *μ*mol/L given 15 min before insulin stimulation) or eNOS by L-NAME (0.5 mmol/L given 15 min before insulin stimulation) markedly inhibited PI3K, Akt, eNOS phosphorylation, and NO production (Fig.11A–D). These results show that dysfunctional PI3K/Akt-mediated eNOS signaling plays an important role in post-MI-associated IR.

## Discussion

In this study, we demonstrated that myocardial infarction causes IR, as well as impaired vasodilation in response to ACh and insulin in aortas, decreased activities of PI3K, Akt, and eNOS, and attenuated insulin-induced PI3K/Akt/eNOS phosphorylation. MI also increases proinflammatory cytokines and p38 MAPK activity. Exercise for 8 weeks following myocardial infarction not only increased the activities of PI3K, Akt, and eNOS, but also elevated phosphorylation of PI3K, Akt, eNOS, and NO production by insulin. Exercise for 8 weeks also normalized the levels of inflammatory cytokines and reduced p38 MAPK activity. This may, help explain how exercise reduces IR. To our knowledge, this is the first study to comprehensively investigate the beneficial effects of exercise on post-MI-induced IR, vascular impairments, and related mechanisms.

### The mechanism of MI-induced IR

Recent studies showed that IR appears in the acute phase of myocardial infarction in nondiabetic patients (Trifunovic et al. 2014). Data showed that IR was associated with an increased risk of incident heart failure among those without diabetes at baseline (Ingelsson et al. 2005; McGuire and Gore 2013). Therefore, finding ways to decrease IR may reduce MI-induced complications and the incidence of heart failure. Previous studies showed that inflammatory cytokines such as IL-1*β*, IL-6, and TNF-*α* are released into blood from MI-injured tissue, and superoxide anion production is increased post-MI; these factors all impair endothelial function (Widder et al. 2004; Nunes et al. 2008; Swirski et al. 2009; Miller et al. 2010). In the present study, MI induced a deterioration of cardiac function and the presence of abnormal insulin and inflammatory cytokines in serum. Numerous studies have shown that there exists a positive correlation between plasma insulin levels and myocardial infarction severity (Bonora et al. 2007; Banerjee et al. 2013; Fukushima et al. 2014). In the present study, insulin in serum was elevated significantly in MI rats compared with Sham rats. The main reasons for this may be associated with post-MI oxidative stress-induced endothelial dysfunction (Wang et al. 2010b; Woth et al. 2013), causing insulin receptor injury and its downstream signaling pathways suppression to lead to IR (Ohta et al. 2011; Fukushima et al. 2014), therefore elevating serum insulin concentrations in post-MI rats. Other studies have shown that myocardial infarction is accompanied by both activated inflammatory response and IR. These inflammatory cytokines might cause the development of IR (Gruzdeva et al. 2013b). Here, we observed not only the degradation of endothelial structure but also the impairment of vasodilation in post-MI aortas. Animal research indicates that MI-induced superoxide production is an important mechanism by which MI impairs vascular function (Zanchi et al. 2008). Moreover, in an animal model of IR, the vascular NO/cGMP/PKG intracellular signaling is impaired due to increased oxidative stress (Doronzo et al. 2011). Other studies strongly suggest that the link between ceramides, IR, and inflammation is related to the inflammatory marker IL-6. Ceramides may contribute to the induction of inflammation involved in IR states that frequently coexists with coronary heart disease (van Gaal et al. 2006). Our study is consistent with previous research; inflammatory cytokines were increased both in serum and in aortic tissue in MI rats, which may be closely involved with IR post-MI, given that the proinflammatory cytokines IL-6 and TNF-*α* are associated with IR (Aires et al. 2013). Meanwhile, TNF-*α*-induced arterial contraction and blocked relaxation (Piepot et al. 2002; Li et al. 2005). Furthermore, TNF-*α* has been reported to stimulate p38 MAPK activity, suggesting a positive feedback loop between p38 MAPK and TNF-*α* (Li et al. 2005). Studies also indicated that post-MI increase in p38 MAPK activity further contributes to vascular dysfunction (Piepot et al. 2002), and p38 MAPK inhibitor SB239063 could improve endothelial dysfunction in post-MI animal model (Widder et al. 2004). Extracellular superoxide dismutase (EC-SOD) knockout was associated with a greater increase in phosphorylated p38 MAPK post-MI (van Deel et al. 2008). Therefore, the activity of p38 MAPK is closely related with endothelial dysfunction. In our present study, the activation of p38 MAPK was markedly elevated, which may be one of the factors in myocardial infarction that cause dysfunction in aortic vasodilation. Moreover, p38 MAPK activation upregulates proinflammatory pathways and is closely associated with IR (Brown et al. 2015); another study showed that there exists cross talk between PI3K/Akt/eNOS signaling and p38 MAPK signaling in aortas (Peng et al. 2010). Our results showed that in post-MI aortas, there is increased phosphorylation of p38 MAPK, and decreased activity of PI3K/Akt/eNOS signaling pathway; these may play an important role in MI's contribution to IR, given that PI3K/Akt pathway has been verified to play a critical role in insulin's metabolic modulation in peripheral insulin-targeted organs (Zhang et al. 2007; Li et al. 2009).

### The mechanism of reduced IR with exercise training

Numerous studies in both humans and animals have demonstrated that exercise training benefits cardiovascular disease and reduces IR (Li et al. 2009; Kim et al. 2014; Ryan et al. 2014). Our previous studies verified that exercise training benefits cardiovascular function in hyperlipidemic and post-MI rats (Wang et al. 2010a,b). However, the mechanisms involved in improving IR due to exercise in post-MI rats remain largely unclear. MI is one of the most important etiologies of heart failure. Importantly, severe MI results in permanent disability or death. Numerous putative mechanisms of exercise-induced cardiovascular protection have been proposed and investigated. These mechanisms include anatomical changes in the coronary arteries, induction of myocardial heat shock proteins, an increase in myocardial cyclooxygenase-2 activity, elevation of endoplasmic reticulum stress proteins, promotion of angiogenesis, and attenuation of left ventricular remodeling in the post-MI failing heart (Petersen and Pedersen 2005; Nunes et al. 2008). Exercise training improves vasomotor function through mechanisms dependent on both increased vascular antioxidant defense and increased NO bioavailability (Kojda and Hambrecht 2005; Petersen and Pedersen 2005; Nunes et al. 2008; Li et al. 2009; Wang et al. 2010b). Exercise training decreases oxidative stress (Zanchi et al. 2008), restores endothelial dysfunction, and activation of insulin signaling further increases the utilization of insulin (Zhang et al. 2007; Li et al. 2009; Wang et al. 2010b), it may be attributed to the decrease in serum insulin that further reduces IR in post-MI rats. eNOS cascade has been shown to play a critical role in vasodilation (Xie and Liu 2009); numerous studies showed increased blood flow due to exercise produces shear stress which is responsible for phosphorylation and activation of eNOS (Zhang et al. 2009; Ramírez-Vélez et al. 2013; Gurovich et al. 2014). Previous studies use culture endothelial cells or isolated blood vessels to demonstrate that mechanical forces and increased perfusion rate induce phosphorylation of Akt/protein kinase B (Akt), protein kinase A (PKA) and/or adenosine monophosphate-activated protein kinase (AMPK) leading to eNOS phosphorylation at serine (S) 1177 (Dixit et al. 2005; Zhang et al. 2007; Laughlin et al. 2008). It has been shown that exercise training mediates blood flow dynamic shear stress, which increases the activation of Akt and AMPK, subsequently contributing to vascular eNOS S1177 phosphorylation (Zhang et al. 2009). In our present study, exercise training increases the activities of PI3K/Akt/eNOS and also increases the phosphorylation of PI3K/Akt/eNOS in response to insulin. Interestingly, exercise training improves vasodilation in response to ACh and insulin by the same pathway of PI3K/Akt-mediated eNOS signaling cascades. Our present studies further indicate that increased expression of TNF-*α* and activation of p38 MAPK were markedly decreased by exercise training, but exercise had no effect on expression of total p38. These results strongly suggest that MI results in significant oxidative stress and increase in proinflammatory cytokines, which may be reversed by exercise training. Recent insight into the mechanisms underlying the beneficial effects of p38 MAPK inhibition suggest that TNF-*α* and IL-6 may be important downstream targets (Kompa et al. 2008; Yin et al. 2008). In our present study, exercise training decreased the expression of TNF-*α* and IL-6 possibly by inhibiting the activities of p38 MAPK. Previous studies indicate that there is cross talk between PI3K/Akt/eNOS signaling pathway and p38 MAPK signaling pathway, PI3K/Akt/eNOS has significant protective effects by preventing capillary leakage through inhibition of p38 MAPK in ventilation-associated mouse lung injury (Peng et al. 2010). Other studies also showed that exercise training attenuates oxidative stress-induced IR through activating the Akt signaling pathway and inhibiting the p38 MAPK signaling pathway in rat skeletal muscle (Vichaiwong et al. 2009). We found that exercise training reduces IR post-MI by the same mechanism that increases PI3K-Akt-eNOS-NO signaling and inhibits p38 MAPK signaling in aortas. This is surprising because the microenvironments of remote organs are markedly different between post-MI and oxidative stress-induced IR. Further investigation is necessary to determine whether exercise-induced activation of PI3K-Akt-eNOS pathway, which directly inhibits p38MAPK activity is involved in exercise-reduced IR in post-MI arteries.

## Conclusion

In summary, the present study demonstrates that under conditions of post-MI-associated IR, increasing insulin levels in the circulation may induce a hypertensive response, and exercise-induced increase in eNOS activation significantly improves insulin sensitivity in aortas. These results suggest that post-MI-associated IR may play an important role in the increased risk for heart failure in MI patients. Therefore, lifestyle interventions that improve insulin sensitivity in aortas, such as physical activity, may have significant value in the prevention and treatment of myocardial infarction and its complications in MI patients.

## Conflicts of interest

No conflicts of interest, financial or otherwise, are declared by the author(s).
